# Pregnancy-associated serum *N*-glycome changes studied by high-throughput MALDI-TOF-MS

**DOI:** 10.1038/srep23296

**Published:** 2016-04-14

**Authors:** Bas C. Jansen, Albert Bondt, Karli R. Reiding, Emanuela Lonardi, Coen J. de Jong, David Falck, Guinevere S. M. Kammeijer, Radboud J. E. M. Dolhain, Yoann Rombouts, Manfred Wuhrer

**Affiliations:** 1 Center for Proteomics and Metabolomics, Leiden University Medical Center, 2300 RC Leiden, The Netherlands; 2 Department of Rheumatology, Erasmus University Medical Center, 3000 CA Rotterdam, The Netherlands; 3 Department of Rheumatology, Leiden University Medical Center, 2300 RC Leiden, The Netherlands; 4 Univ. Lille, CNRS, UMR 8576, UGSF, Unité de Glycobiologie Structurale et Fonctionnelle, F 59 000 Lille, France

## Abstract

Pregnancy requires partial suppression of the immune system to ensure maternal-foetal tolerance. Protein glycosylation, and especially terminal sialic acid linkages, are of prime importance in regulating the pro- and anti-inflammatory immune responses. However, little is known about pregnancy-associated changes of the serum *N*-glycome and sialic acid linkages. Using a combination of recently developed methods, *i.e.* derivatisation that allows the distinction between α2,3- and α2,6-linked sialic acids by high-throughput MALDI-TOF-MS and software-assisted data processing, we analysed the serum *N*-glycome of a cohort of 29 healthy women at 6 time points during and after pregnancy. A total of 77 *N*-glycans were followed over time, confirming in part previous findings while also revealing novel associations (*e.g.* an increase of FA2BG1S1(6), FA2G1S1(6) and A2BG2S2(6) with delivery). From the individual glycans we calculated 42 derived traits. With these, an increase during pregnancy and decrease after delivery was observed for both α2,3- and α2,6-linked sialylation. Additionally, a difference in the recovery speed after delivery was observed for α2,3- and α2,6-linked sialylation of triantennary glycans. In conclusion, our new high-throughput workflow allowed the identification of novel plasma glycosylation changes with pregnancy.

Glycosylation is the most common post-translational modification, and is one of the main factors regulating innate and adaptive immune responses^1^. Changes in glycosylation have been associated with various physiological (*e.g.* age and sex) and pathological conditions (*e.g.* infection, autoimmune disease and cancer)^2^,^3^,^4^. Disease-associated changes in glycosylation have been reported for various serum proteins, including acute phase proteins (*e.g.* α1-acid glycoprotein (AGP), hemopexin and haptoglobin (HPT)) and immunoglobulins, produced by liver cells and plasma B-cells respectively^5^,^6^. In particular, the levels of α2,6-linked sialylation and galactosylation of the *N*-glycan in the immunoglobulin G (IgG) fragment crystallisable (Fc)-domain have been correlated with the severity of various diseases including rheumatoid arthritis (RA) and inflammatory bowel disease^3^,^6^,^7^,^8^,^9^. Additionally, sialylation and galactosylation are known to regulate the inflammatory properties of IgG, by modifying its tertiary structure and interaction with receptors such as the Fc-receptors, C1q and DC-SIGN^10^,^11^.

Various changes in the glycosylation of serum proteins, including IgG and α1-antitrypsin (AAT), have been observed during pregnancy^3^,^12^,^13^. Pregnancy poses major immunological challenges due to genetic and antigenic differences between mother and child^14^. Partial suppression of the maternal immune system is required to ensure tolerance of the foetus, and one may hypothesize that glycosylation plays a role in this^15^. Pregnancy leads to an increase in IgG Fc-linked *N*-glycan galactosylation and sialylation, two features with a proven anti-inflammatory role (regarding sialylation only with α2,6- and not α2,3-linked sialic acids)^3^,^16^,^17^.

Protein glycosylation changes studied in individual proteins show highly specific correlations, but such targeted approaches may be blind to biologically important changes originating from other glycoproteins within the serum pool. As an example, the analysis of the total serum *N*-glycome (TSNG) allows the observation of sialyl-Lewis antigens (sialyl-Lewis X or sialyl-Lewis A), which are well-known glycan epitopes involved in immunity and inflammation^18^,^19^. In contrast, targeted analysis of IgG glycosylation would miss the observation of a sialyl-Lewis motif, for the simple reason that none of the IgG glycoforms contain this epitope^20^.

The profiling of a complex sample, such as TSNG, can be performed using a combination of separation and detection methods. For example, HILIC (hydrophilic interaction liquid chromatography) may be combined with either CGE-LIF (capillary gel electrophoresis - laser induced fluorescence) or HPLC-FL (high-performance liquid chromatography - fluorescence)^12^,^21^. Alternatively, glycans can be permethylated and measured by LC-MS (liquid chromatography - mass spectrometry)^22^. A different technique extensively used for high-throughput profiling is MALDI-TOF-MS (matrix-assisted laser desorption/ionization - time-of-flight - mass spectrometry), which allows for the acquisition of a glycan spectrum within seconds, while LC based methods generally require much more time^23^,^24^. Moreover, technical challenges posed by the instability and ionization bias observed for sialylated glycans have been successfully overcome by permethylation as well as by selective stabilisation of sialic acids by *e.g.* ethyl esterification^25^,^26^,^27^,^28^,^29^,^30^. The latter method introduces a mass difference between α2,3- and an α2,6-linked sialic acids, allowing their discrimination by mass spectrometry, and is well suited for high-throughput *N*-glycan profiling using MALDI-TOF-MS^27^.

An important issue that arises when analysing larger sample sets by MALDI-TOF-MS is data processing, which is often the rate-limiting step. The recently developed processing software package, MassyTools, offers a valid solution to this problem by rapidly integrating a predefined list of analytes from any number of mass spectra^31^. Additionally, MassyTools calculates a wide variety of spectral and analyte quality criteria that allow for an efficient and reliable automated curation of (clinical) data.

In this study, we applied high-throughput MALDI-TOF-MS profiling together with a newly developed, automated data processing tool to the TSNG analysis of a longitudinal cohort of healthy women (n = 29) encompassing time points during pregnancy and after delivery^31^. Pregnancy-associated glycosylation changes have previously been studied in this cohort for specific proteins such as IgG, AAT and immunoglobulin A (IgA), as well as for serum glycans using CGE-LIF methodology^12^,^32^. The use of derived traits allowed us to obtain additional biological insight, as the calculated derived traits are mainly based on single enzymatic step monosaccharide modifications. Furthermore, the method allowed us to identify more glycans with a higher confidence, including differentiation of some isomers, than with the previously mentioned CGE-LIF based study. The utilised methods for sample preparation, as well as data processing, proved to be fast and reliable. Data analysis revealed previously unnoticed *N*-glycomic changes with differential dynamics in sialylation and abundance of triantennary glycans.

## Results

**Figure 1:**
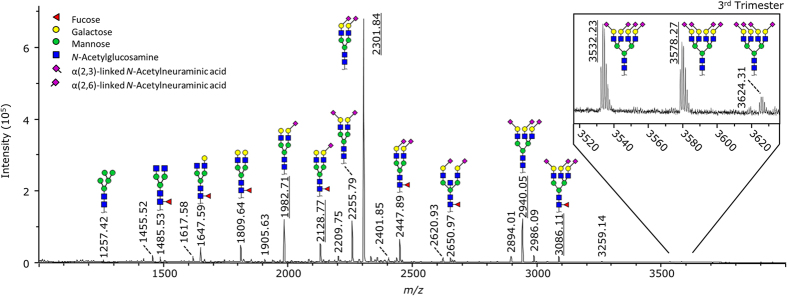
Mass spectrometric serum *N*-glycome profile. Average reflectron positive-ion mode MALDI-TOF-MS spectrum of ethyl esterified released serum glycans, derived from the 3^rd^ trimester samples of the 29 participants. Calibrants are underlined. The shown *N*-glycan structures are based on literature and the observed mass^27^. A more detailed and annotated spectrum is provided as Supplementary Fig. S1.

**Figure 2:**
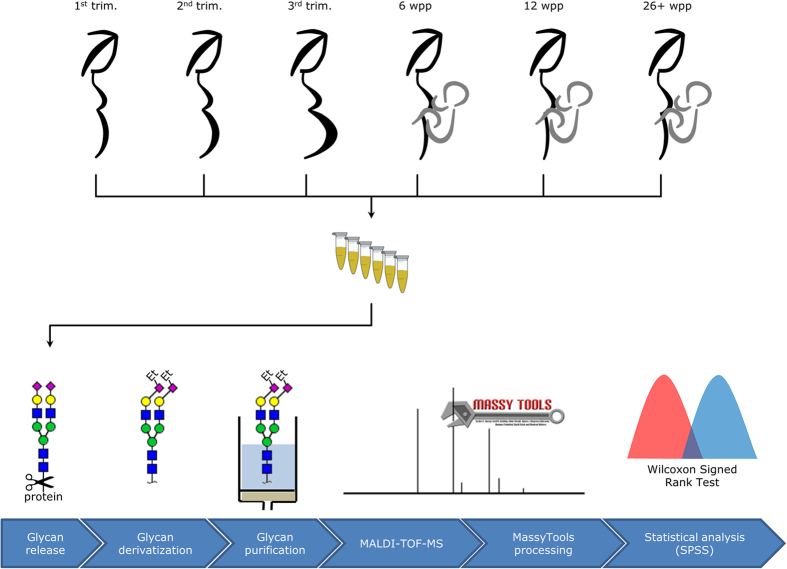
Glycomics workflow. The workflows illustrates the sample collection scheme, as well as the steps of the glycomics workflow: *N*-glycans were enzymatically released from serum, ethyl esterified and finally purified by HILIC-SPE^27^. Samples were then measured by MALDI-TOF-MS. Lastly, the resulting profiles were processed with MassyTools and SPSS^31^.

The TSNG profiles of 29 healthy Caucasian women, screened at three time points during pregnancy and three time points after delivery, were analysed using MALDI-TOF-MS after enzymatic glycan release and linkage-specific sialic acid derivatisation (Fig. 1; Supplementary Fig. S1)^27^. The entire workflow is displayed in Fig. 2. A table showing all integration and curation results is included in the Supplementary Table S1. A set of 77 *N*-glycan compositions were consistently detected and thus quantified in the glycan profiles acquired by MALDI-TOF-MS (Supplementary Table S2), from which 42 derived traits were calculated based on the compositional features (Supplementary Table S3). The retained compositions included 56 sialylated structures, of which 31 contained α2,3-linked and 45 contained α2,6-linked sialylation. Furthermore, we extracted 5 oligomannosidic, 7 hybrid, 32 fucosylated and 13 bisected compositions. All calculated glycosylation traits were compared pairwise using a Wilcoxon signed-rank test to highlight differences between time points (Table 1; Supplementary Table S4). Additionally, all (77) individual glycan traits were compared pairwise using a Wilcoxon signed-rank test (Supplementary Table S5). A summary of the individual glycan results can be found in the Supplementary Table S6. Bonferroni correction was used to address the multiple testing problem, by adjusting the significance threshold (*e.g.* a significance level α < 0.0012 for 42 tests per time point)^33^.

### TSNG changes during pregnancy

**Figure 3:**
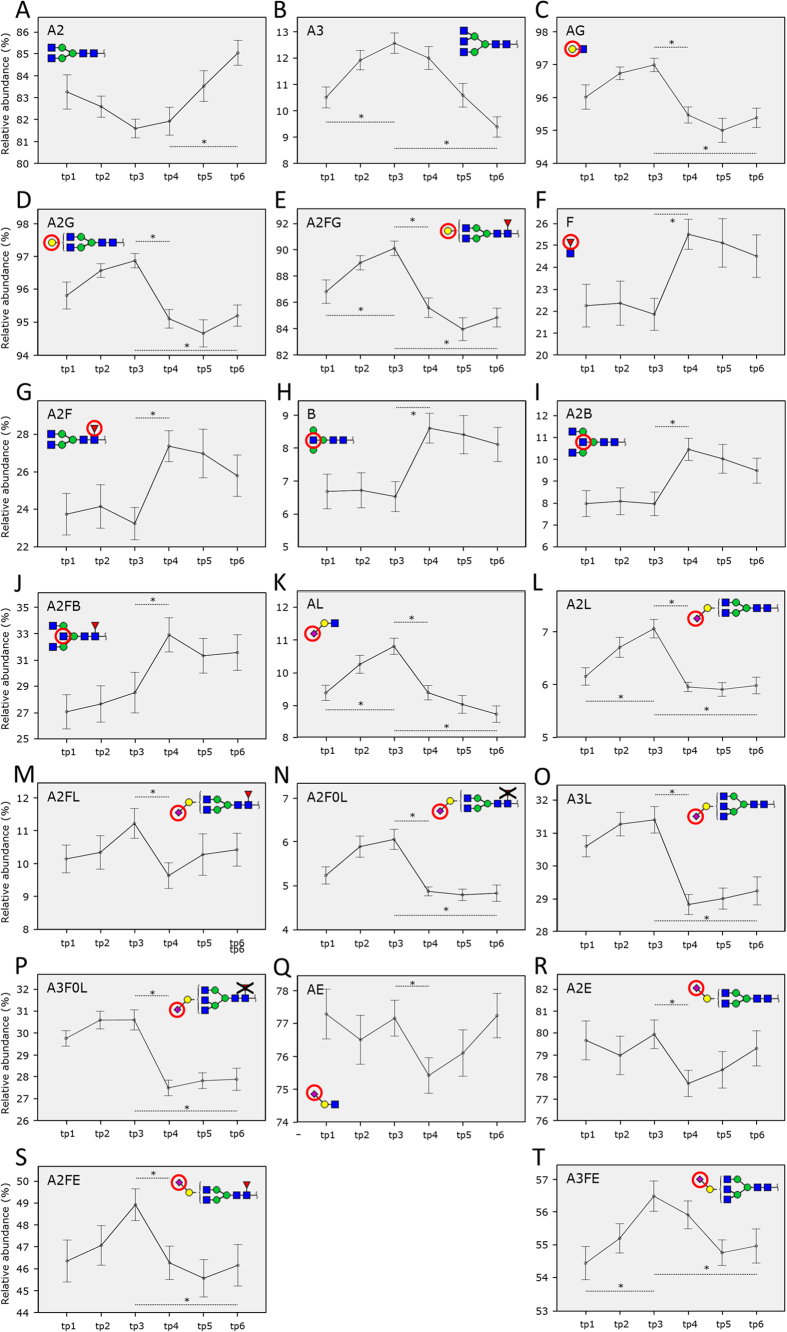
Pregnancy-associated serum *N*-glycosylation changes. The graphs represent the relative abundances of significantly changing glycosylation traits: (**A**) diantennary glycans, (**B**) triantennary glycans, (**C**) galactosylation of all glycans, (**D**) galactosylation of diantennary glycans, (**E**) galactosylation of diantennary fucosylated glycans, (**F**) fucosylation of all glycans, (**G**) fucosylation of diantennary glycans, (**H**) bisection of all glycans, (**I**) bisection of diantennary glycans, (**J**) bisection of diantennary fucosylated glycans, (**K**) α2,3-linked sialylation of complex glycans, (**L**) α2,3-linked sialylation of diantennary glycans, (**M**) α2,3-linked sialylation of diantennary fucosylated glycans, (**N**) α2,3-linked sialylation of diantennary non-fucosylated glycans, (**O**) α2,3-linked sialylation of triantennary glycans, (**P**) α2,3-linked sialylation of triantennary non-fucosylated glycans, (**Q**) α2,6-linked sialylation of complex glycans, (**R**) α2,6-linked sialylation of diantennary glycans, (**S**) α2,6-linked sialylation of diantennary fucosylated glycans, and (**T**) α2,6-linked sialylation of triantennary fucosylated glycans. The minimum glycoform structure is displayed in each panel with a red circle indicating the feature of interest. The values listed for galactosylation and sialylation are per antenna. The graph shows the standard error for each time point. Additionally, significant differences between time points are marked with a dashed line and an asterisk. Formulae used for glycosylation trait calculation are given in Supplementary Table S2.

When comparing TSNG glycosylation features between the 1^st^ and 3^rd^ trimester (trim) of pregnancy, statistical analyses revealed a rise in the level of triantennary glycans (A3; 10.5% to 12.6%; *p*  =  0.0005; Fig. 3B) as well as complex changes in sialylation patterns: we observed increased levels of α2,3-linked sialylation of complex glycans (AL; 9.4% to 10.8%; *p* = 0.0003; Fig. 3K), especially in diantennary glycans (A2L; 6.2% to 7.1%; *p* = 0.0004; Fig. 3L). Furthermore, there was an increase of α2,6-linked sialylation in triantennary fucosylated glycans (A3FE; 54.4% to 56.5%; *p* = 0.0001; Fig. 3T). The increase of sialylation in diantennary glycans is in line with an increase of galactosylation of diantennary fucosylated glycans (A2FG; 86.8% to 90.1%; *p* < 0.0001; Fig. 3E). However, we observed only a trend towards an increase in the related traits of galactosylation of all complex type glycans (AG; 96.0% to 97.0%; *p* = 0.0019; Fig. 3C) and galactosylation of diantennary glycans (A2G; 95.8% to 96.9%; *p* = 0.0014; Fig. 3D). Interestingly, we also observed a trend towards an increase in antenna fucosylation, indicative of the presence of a sialyl-Lewis epitope, although this was found non-significant after multiple testing correction (0.07% to 0.09%; *p* = 0.0019; data not shown).

### TSNG changes during the short term recovery

During short term recovery after delivery (comparison between 3^rd^ trim and 6 weeks postpartum (WPP)) glycosylation changes appeared more pronounced than during either pregnancy or long term recovery. Galactosylation levels were overall decreased (AG; 97.0% to 95.5%; *p* = 0.0001; Fig. 3C), a finding directly related to decreased levels of galactosylation of diantennary glycans (A2G; 96.9% to 95.1%; *p* < 0.0001; Fig. 3D) and in particular decreased levels of galactosylation of diantennary fucosylated glycans (A2FG; 90.1% to 85.6%; *p* < 0.0001; Fig. 3E). Additionally, we found decreased levels of α2,3-linked sialylation: a decrease of α2,3-linked sialylation of complex glycans was observed (AL; 10.8% to 9.4%; *p* = 0.0001; Fig. 3K), in particular for α2,3-linked sialylation of diantennary glycans (A2L; 7.1% to 6.0%; *p* < 0.0001; Fig. 3L), both fucosylated (A2FL; 11.2% to 9.6%; *p* = 0.0001; Fig. 3M) and non-fucosylated (A2F0L; 6.1% to 4.9%; *p* = 0.0001; Fig. 3N). Additionally, the observed decrease in α2,3-linked sialylation of triantennary glycans (A3L; 31.4% to 28.8%; *p* = 0.0001; Fig. 3O) can be attributed to a decrease in α2,3-linked sialylation of triantennary non-fucosylated glycans (A3F0L; 30.6% to 27.5%; *p* < 0.0001; Fig. 3P). Besides a decrease in α2,3-linked sialylation, we also observed a decrease in α2,6-linked sialylation of complex glycans (AE; 77.2% to 75.4%; *p* = 0.0007; Fig. 3Q). This can be traced to the diantennary glycans (A2E; 80.0% to 77.7%; *p* = 0.0004; Fig. 3R) and further to diantennary fucosylated glycans (A2FE; 48.9% to 46.3%; *p* = 0.0010; Fig. 3S).

Beyond decreasing levels of galactosylation and sialylation, we also observed increases in bisection and fucosylation. Increased levels of fucosylation (F; 21.9% to 25.5%; *p* < 0.0001; Fig. 3F) were partly due to an increase in fucosylation of diantennary glycans (A2F; 23.3% to 27.4%; *p* < 0.0001; Fig. 3G). Increased levels of bisection (B; 6.5% to 8.6%, *p* < 0.0001; Fig. 3H) could be further assigned to bisection of diantennary glycans (A2B; 8.0% to 10.5%; *p* < 0.0001; Fig. 3I) and to diantennary fucosylated glycans (28.5% to 32.9%; *p* < 0.0001; Fig. 3J).

### TSNG changes during the long term recovery

Long term recovery (comparison between 3^rd^ trim and 26+ WPP samples) showed changes in the types of glycans, as demonstrated by a decrease of triantennary glycans (A3; 12.6% to 9.4%; *p* = 0.0001; Fig. 3B) and an increase of diantennary glycans (A2; 81.6% to 85.0%; *p* = 0.0004; Fig. 3A). Decreased levels of galactosylation were observed for all glycans (AG; 97.0% to 95.4%; *p* = 0.0001; Fig. 3C), and in particular for diantennary glycans (A2G; 96.9% to 95.2%; *p* = 0.0001; Fig. 3D), especially fucosylated (A2FG; 90.1% to 84.9%; *p* < 0.0001; Fig. 3E). We also identified decreased levels of α2,3-linked sialylation of complex glycans (AL; 10.8% to 8.7%; *p* = 0.0001; Fig. 3K), especially in diantennary glycans (A2L; 7.1% to 6.0%; *p* = 0.0002; Fig. 3L) and diantennary non-fucosylated glycans (A2F0L; 6.1% to 4.8%; *p* = 0.0003; Fig. 3N). Triantennary structures also showed a decrease in levels of α2,3-linked sialylation (A3L; 31.4% to 29.2%; *p* = 0.0011; Fig. 3O), especially in non-fucosylated structures (A3F0L; 30.6% to 27.9%; *p* = 0.0010; Fig. 3P). Interestingly, the levels of α2,6-linked sialylation of complex glycans were not changed (77.2% in both 3^rd^ trim and 26+ WPP samples). However, the levels of α2,6-linked sialylation of diantennary fucosylated glycans (A2FE; 48.9% to 46.2%; *p* = 0.0008; Fig. 3S) and triantennary fucosylated glycans (A3FE; 56.5% to 55.0%; *p* = 0.0004; Fig. 3T) were decreased. Additionally, we also observed a non-significant decrease in antenna fucosylation (0.09% to 0.07%; *p* = 0.0033; data not shown), indicative of the reduction of a sialyl-Lewis epitope.

## Discussion

The total serum *N*-glycome of a set of 174 serum samples from 29 individuals taken at 3 time points during pregnancy and 3 time points after delivery was analysed using a recently developed glycan derivatisation technique able to differentiate between α2,3- and α2,6-linked *N*-acetylneuraminic acids (sialic acids)^27^. This was used in combination with high-throughput MALDI-TOF-MS detection and a high-throughput data extraction software package^31^. The sample set has previously been studied by CGE-LIF, showing an increase in highly sialylated di-, tri- and tetraantennary glycans and a decrease in diantennary structures with zero or one sialic acid, which was confirmed in the current study^12^. While CGE-LIF potentially differentiates more isomers than the method applied herein, the usefulness of the separation is often compromised by the overlap of glycan species due to a necessary compromise between resolution and throughput^34^. MALDI-TOF-MS normally cannot separate isomers without fragmentation. However, by combining it with a recently developed sialic acid derivatisation method, we did obtain information on the sialic acid linkage^27^. Furthermore, we applied statistics to the derived glycosylation traits instead of individual glycans, as derived traits offer a higher robustness and more directly provide insights into changes in glycosylation pathways^35^. The CGE-LIF study could not use derived traits as the potential overlap of glycan species with diverging properties compromises the construction of derived traits with unambiguous biological meaning. We compared our results with the CGE-LIF study at the individual glycan level. The comparison shows that we reproduced many of the previously measured CGE-LIF results and additionally found many novel findings, including glycosylation changes during pregnancy (1^st^ trimester *vs.* 3^rd^ trimester), and that various additional glycoforms, including isomers, change with delivery (3^rd^ trimester *vs.* 6 WPP) and long term recovery (3^rd^ trimester *vs*. 26+ WPP; Supplementary Table S7). For instance, we observed an increase of H5N5E2, H4N5F1E1 and H4N4F1E1 with delivery, while all of these glycans were not detected using CGE. However, there were also results that we were not able to reproduce, such as an increase in H5N4 with the delivery. In the previous paper the responsible CGE-LIF peak had been assigned to two potential glycoforms, namely H5N4 and H4N5F1. In the current study by MALDI-TOF-MS, however, we did not identify the H5N4, while the H4N5F1 peak we did detect matched the CGE-LIF data. Therefore, in the light of the mass spectrometric data, one possibility would be that the previously detected CGE-LIF peak did not consist of both H5N4 and H4N5F1, but rather of only H4N5F1.

The applied MALDI-TOF-MS method showed no changes in either oligomannosidic or hybrid glycans, confirming previous results by CGE-LIF^12^. However, a variety of glycosylation changes was observed in complex glycans over time (Fig. 3). The glycosylation changes in serum are caused in part by changes in protein-specific glycosylation and in part by changes in glycoprotein levels due to pregnancy (*e.g.* decreasing levels of AAT and IgG)^3^,^12^,^13^,^32^,^36^.

**Figure 4:**
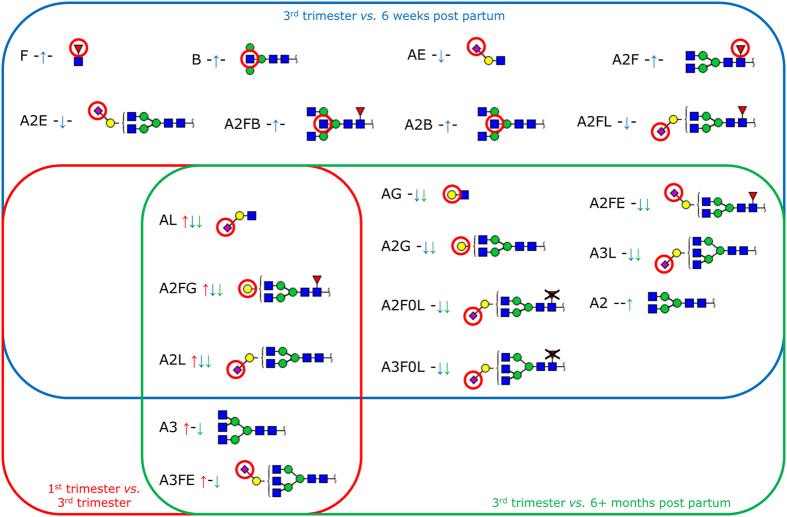
Overview on serum glycosylation changes during pregnancy, with delivery and recovery. Glycosylation traits that significantly change throughout pregnancy are shown in a Venn diagram. Traits are clustered based on when they showed a change, *i.e.* pregnancy (1^st^ trimester versus 3^rd^ trimester), short term recovery (3^rd^ trimester versus 6 WPP) and the long term recovery (3^rd^ trimester versus 26+ WPP). The direction of the change is marked by arrows: an upwards arrow indicates that a trait is increased while a downwards arrow indicates that a trait is decreased. The minimum glycoforms structure is displayed next to each trait with a red circle indicating the feature of interest for the respective trait. A similar figure for all the glycoforms is included as Supplementary Fig. S3.

We observed an increase in galactosylation of diantennary fucosylated glycans during pregnancy, followed by a sharp decrease with delivery (A2FG; Fig. 4). In addition, we observed a trend for increasing α2,6-linked sialylation of diantennary fucosylated glycans (A2FE; 46.4% to 48.9%), but this was not significant after multiple testing correction (*p* = 0.0141). IgG is one of the most prominent serum proteins carrying diantennary fucosylated glycans, of which only a small fraction contains α2,6-linked sialic acids^6^. The levels of IgG decrease with pregnancy from 9.5 mg/mL (7–17 weeks of pregnancy) to 7.8 mg/mL (34–38 weeks), resulting in an increase in the contribution of other proteins, such as IgM, to the A2FE trait^36^. Together with the known increase in α2,6-linked sialylation of IgG *N*-glycans during pregnancy this results in a major contribution of IgG-related changes to the observed increase in A2FE^3^,^32^. The fact that this increase is hardly visible in TSNG analysis may indicate that changes in other glycoproteins, with respect to concentration and glycosylation, counteract the IgG-associated changes. Interestingly, IgM shows an opposite trend with pregnancy with its levels increasing from 1.2 mg/mL during 7–17 weeks of pregnancy to 1.46 mg/mL during 34–38 weeks of pregnancy^36^. While IgM is known to carry diantennary core-fucosylated *N*-glycans with rather high levels of α2,6-linked sialylation, its glycoform-related pregnancy-associated glycosylation changes are not known^6^,^37^. Hence, changes in IgM glycosylation over the course of pregnancy should be investigated.

Our results show specific longitudinal glycosylation changes in serum triantennary glycans. The levels of triantennary structures and their α2,3- or α2,6-linked sialylated variants show an increase during pregnancy (1^st^ trimester to 3^rd^ trimester) followed by a decrease after delivery (3^rd^ trimester to 6 WPP). This could reflect changes in the protein concentrations or glycosylation of abundant glycoproteins that contain triantennary glycans, such as AAT, HPT and AGP^6^,^38^,^39^,^40^. The protein concentration of AGP remains stable throughout pregnancy, but whether the glycosylation levels change with pregnancy is unknown^36^. In contrast, AAT and HPT both increase in concentration during pregnancy and could therefore be responsible for the observed increase in triantennary glycans^36^. The main function of AAT is to protect tissue from enzymatic attacks^41^. One may speculate that an increase in AAT could be attributed to a mechanism where the developing embryo and its supporting structures (placenta and endometrium) are protected from enzymatic attacks by the immune system of the mother. Interestingly, the previously mentioned CGE-LIF study on the same cohort also showed increased levels of triantennary glycans on AAT during pregnancy^12^. Therefore, it is likely that a combination of the increasing concentration of AAT and the increase in triantennary glycans of AAT make a major contribution to the observed increase in triantennary glycans in the TSNG.

The linkage of the sialic acid is of major importance to determine if a sialyl-Lewis epitope is present on a glycan. A sialyl-Lewis X or sialyl-Lewis A epitope requires the presence of both a fucose (α1,3-linked for sialyl-Lewis X or α1,4-linked for sialyl-Lewis A) and an α2,3-linked sialic acid^42^. In diantennary glycans the fucose is mostly present in the core, while in tri- and tetraantennary glycans it is generally on the antenna, resulting in a sialyl-Lewis X or A epitope^6^. However, we did not observe any changes in relation to the percentage of sialyl-Lewis epitopes during and after pregnancy. Our results do show changes in α2,6-linked sialylation of fucosylated triantennary glycans (A3FE; Fig. 3T). In addition, we found changes in α2,3-linked sialylation of triantennary glycans (A3L; Fig. 3O), but this could be further narrowed down to changes in α2,3-linked sialylation of non-fucosylated triantennary glycans (A3F0L; Fig. 3P). Therefore, it is apparent that there are no changes related to sialyl-Lewis epitopes, or that the changes are obscured by changing glycoprotein levels, such as AAT^36^.

Many physiological processes are involved in the postpartum recovery of the mother. Homeostasis needs some time to re-establish and this is reflected by the changes in the serum glycome. Some traits rapidly change (between the 3^rd^ trimester and 6 WPP) and then stabilise (similar levels at 6 and 26+ WPP), while variations in others emerge only later in time (significant at 26+ WPP; Fig. 4). For IgG it has been shown for RA patients that the glycosylation restores postpartum within the first 6 WPP to a similar level as before the pregnancy^3^. Accordingly, we observed in the current study a similar time course for the galactosylation of diantennary fucosylated glycans. Similarly, also α2,3-linked sialylation of both di- and triantennary glycans, clearly non-IgG related, are examples of fast responses^32^. However, other traits did not recover within the first 6 WPP but only changed after 26 weeks or more, namely the number of antennae on complex type glycans and α2,6-linked sialylation of triantennary fucosylated glycans (Fig. 4).

A major advantage of our study design is that patient samples are collected from a longitudinal cohort, thereby reducing the impact of biological variance. Consequently, our results can be attributed purely to the pregnancy-associated changes. However, a minor drawback is represented by isomeric glycan structures that we were unable to resolve. Typical examples include H6N5E2, which could be either a triantennary glycan or a diantennary glycan with a LacNAc repeat, and H5N5, which could be either a truncated triantennary glycan or a bisected diantennary glycan. Consequently, the use of compositional information to assign a derived trait may to a certain extent lead to glycan misclassifications. Therefore, the derived traits are calculated based on the most common structure assigned to a distinct composition^6^. In addition, many glycomic studies deal with age, sex and medication as major confounders, but none of these are applicable here as the study encompassed only healthy women of roughly the same age (32.1 years, SD ± 4.4 years)^4^. However, we did not take into account other confounders such as breastfeeding, as the resulting group sizes would lead to low statistical power. Despite the limitations of our study, the results should be indicative of changes that occur during and after pregnancy, as they agree with previous literature^12^,^32^.

In conclusion, we observed changes in galactosylation, sialylation and antennarity of the TSNG during pregnancy. Furthermore, differences were observed in the galactosylation, bisection, fucosylation, sialylation and antennarity of the TSNG after delivery. Lastly, a difference in the recovery speed was observed between α2,3- and α2,6-linked sialylation of triantennary glycans. However, the biological relevance of the observed changes in the sialylation of triantennary structures and their linkages is at present unknown and will require further study.

## Methods

### Materials

Sodium dodecyl sulphate (SDS), ethanol and trifluoroacetic acid were purchased from Merck (Darmstadt, Germany). Nonidet P-40 substitute (NP-40), super-DHB (9:1 mixture of 2,5-dihydroxybenzoic acid and 2-hydroxy-5-methoxybenzoic acid)^43^, sodium hydroxide (NaOH) and 1-hydroxybenzotriazole hydrate (HOBt) were acquired from Sigma-Aldrich (Steinheim, Germany). 1-Ethyl-3-(3-(dimethylamino)propyl)carbodiimide hydrochloride (EDC) was acquired from Fluorochem (Hadfield United Kingdom). Peptide-*N*-glycosidase F (PNGase F) was supplied by Roche Diagnostics (Mannheim, Germany). Cotton thread was bought from Pipoos (Utrecht, The Netherlands). HPLC SupraGradient acetonitrile (ACN) was acquired from Biosolve (Valkenswaard, The Netherlands). Peptide calibration standard and an AnchorChip MALDI target plate were purchased from Bruker Daltonics (Bremen, Germany). Pooled plasma from 20 healthy human donors was purchased from Affinity Biologicals (Ancaster, Canada) and used as control sample. Lastly, water was purified with a Purelab Ultra from Elga LabWater (Ede, The Netherlands).

### Samples

The sera of 29 healthy Caucasian women (mean age 32.1 years, SD ± 4.4) were obtained at three time points during pregnancy (1^st^, 2^nd^ and 3^rd^ trimester), and three time points after delivery (6, 12 and 26+ WPP). Sera were collected within the framework of the nationwide (The Netherlands) prospective Pregnancy-associated Amelioration of Rheumatoid Arthritis (PARA) study, designed to investigate the improvement of RA during pregnancy^44^. The study was in compliance with the Helsinki Declaration, informed consent was obtained from all subjects, and the study was approved by the Erasmus University Medical Center Ethics Review Board. A total of 174 PARA (6 ^*^ 29) serum samples from the healthy control group of the PARA cohort were analysed. Furthermore, 10 replicates of a commercially available pooled plasma sample of healthy volunteers were used as a technical control, yielding an RSD of the main peak (H5N4E2) of 7% (Supplementary Fig. S4). The use of derived traits reduced the technical variation, for instance the RSD of the derived trait ‘galactosylation of all glycans’ (AG) was found to be 0.8% for the technical control.

### Sample preparation

In order to analyse the TSNG of healthy women during pregnancy, 10 μL serum was mixed with 20 μL 2% SDS, prior to denaturation by 10 min incubation at 60 °C. *N*-glycans were released by addition of 20 μL release mixture (1:1 4% NP-40: 5x PBS) containing 1 U PNGase F, followed by overnight incubation at 37 °C. Of the 50 μL released glycans, 1 μL was derivatised by ethyl esterification as reported in literature^27^. Briefly, the 1 μL sample was added to 20 μL 250 mM EDC + 250 mM HOBt in pure ethanol and incubated for 1 h at 37 °C. Following derivatisation, the glycans were purified by hydrophilic interaction liquid chromatography (HILIC)-solid phase extraction (SPE) using cotton thread tips, as described previously^32^. The cotton material, packed into pipette tips, was pre-conditioned by pipetting three times 20 μL water, followed by equilibration with three times 20 μL 85% ACN. The samples were mixed with 20 μL ACN and then pipetted through the pre-equilibrated cotton material extensively. The tips were sequentially washed three times with 20 μL 85% ACN + 1% trifluoroacetic acid and three times with 85% ACN. Glycans were eluted in 10 μL water. Afterwards, 1 μL of the TSNG SPE eluate was applied to an AnchorChip MALDI target plate, mixed with 1 μL of 5 mg/mL super-DHB in 50% aqueous ACN containing 1 mM NaOH, and dried at room temperature.

### Data acquisition

Derivatised glycan samples were measured using the reflectron positive ion mode of an Ultraflextreme MALDI-TOF-MS (Bruker Daltonics), equipped with a Smartbeam-II laser, and operated by flexControl 3.4 build 135. Prior to the measurement, external calibration was performed using a peptide calibration standard. Ions were accelerated at 25 kV with 140 ns delayed extraction. Per sum spectrum, a total of 20,000 shots were accumulated between *m/z* 1,000 and *m/z* 5,000.

### MassyTools data processing

Using the flexAnalysis Batch Process program (3.3, build 65), the obtained spectra were exported into a text-based format ((x,y) file) consisting of an *m/z* value coupled with an intensity value per line, separated by a tab. In order to perform processing by MassyTools; software freely available at https://github.com/Tarskin/MassyTools), we applied the ‘free’ reducing end mass modifier and ‘sodium’ charge carrier as general parameters^31^. Additionally, a single letter code was utilized by the program to refer to monosaccharides: H for hexose, N for *N*-acetylhexosamine, F for fucose, E for an α2,6-linked *N*-acetylneuraminic acid and L for an α2,3-linked *N*-acetylneuraminic acid.

Internal calibration was performed using a minimum of 5 out of a list of 7 potential calibrants passing a signal-to-noise (S/N) ratio threshold of 9. Potential calibrants were H5N4E1 (1,982.708 Da), H5N4F1E1 (2,128.766 Da), H5N4E2 (2,301.835 Da), H6N5E2L1 (2,940.052 Da), H6N5F1E2L1 (3086.110 Da), H7N6E1L3 (3,532.227 Da) and H7N6E2L2 (3,578.269 Da; [M + Na]^+^ throughout). Additionally, we required a minimum of 4 calibrants between *m/z* 1,000 and *m/z* 2,333 as well as a minimum of 1 calibrant between *m/z* 2,333 and *m/z* 3,667. The program was allowed to detect calibrants within a window of *m/*z 0.4 around the exact mass. The calibration was assessed by evaluating the mass error of the major calibrant glycan (H5N4E2) and major non-calibrant glycan (H5N4E1L1) for the technical control samples. H5N4E2 showed an average mass error of −0.3 ppm, SD ± 1.9 ppm while H5N4E1L1 showed an average mass error of −3.1 ppm, SD ± 3.0 ppm.

Extraction was performed using a ± *m/z* 0.49 window. A list of 109 analytes was used for initial extraction (Supplementary Table S1). The software was allowed to use an *m/z* region of 15 around the analyte to identify the background area and noise values. Subsequently, spectral and analyte curation was performed. Firstly, the “fraction of spectrum in analytes” and “fraction of analyte area above S/N cut-off” parameters were used for spectral curation. The spectral quality criteria of all spectra were found to be within three times SD of the average, therefore all spectra were kept for further analysis. Secondly, a quality control (QC) parameter and a S/N cut-off were used to curate the analyte list: analytes with an average QC value above 1.8 × 10^−4^ or an average S/N of below 6 were discarded, while analytes with an average QC value below 1.8 × 10^−4^ and an average S/N of above 6 (Supplementary Fig. S2) were retained. A total of 77 analytes passed these quality criteria and were used for further analysis.

### Derived traits calculation & statistical analysis

Glycans were grouped according to their compositional features. All derived traits are based on single enzymatic steps (*e.g.* AG for the attachment of a galactose to an *N*-acetylhexosamine). The total set of 42 calculated derived traits can be found in the Supplementary Table S2. Furthermore, statistical analysis was performed using IBM SPSS Statistics 20 (IBM, New York, USA). All derived traits were tested for normality using a Shapiro-Wilk test^45^. As part of the derived traits were not normally distributed, a Wilcoxon signed-rank test was used to test pairwise differences observed in the cohort^46^. All 42 traits were tested by comparing the 1^st^ trimester with the 3^rd^ trimester (pregnancy), the 3^rd^ trimester with 6 WPP (short term recovery) and, lastly, the 3^rd^ trimester with 26+ WPP (long term recovery). Bonferroni correction was used to address the multiple testing problem, by adjusting the significance threshold (significance level α < 0.0012 for 42 tests per time point)^33^.

## Additional Information

**How to cite this article**: Jansen, B. C. *et al.* Pregnancy-associated serum *N*-glycome changes studied by high-throughput MALDI-TOF-MS. *Sci. Rep.* **6**, 23296; doi: 10.1038/srep23296 (2016).

## Supplementary Material

Supplementary Information

Supplementary Tables

## Figures and Tables

**Table 1:**
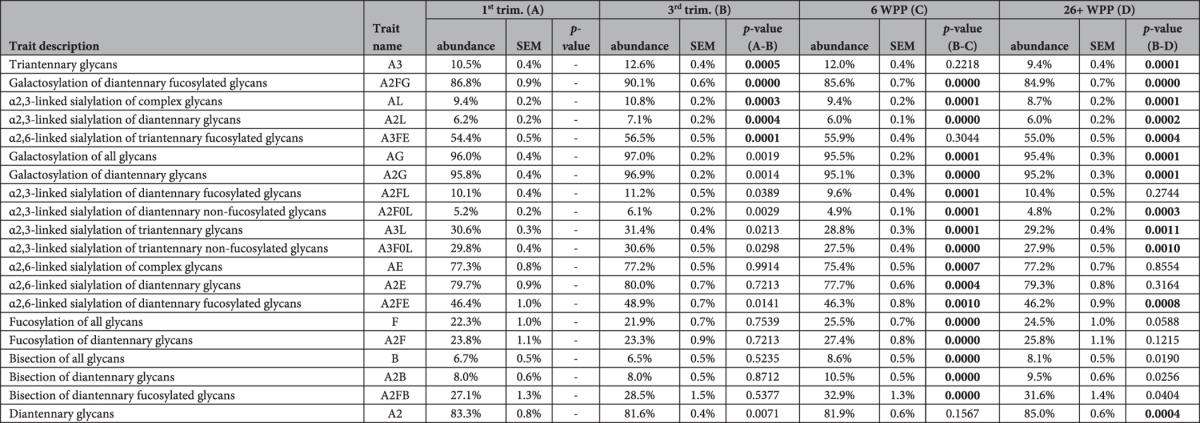
Pregnancy related changes in glycosylation.

The table displays the significant changes of derived glycan traits throughout pregnancy. The shown abundance values are the mean followed by the standard error of the mean. Comparisons were performed as follows: 1^st^ trimester (trim) *vs.* 3^rd^ trim, 3^rd^ trim *vs.* 6 weeks post pregnancy (WPP), and 3^rd^ trim *vs.* 26 WPP. A Wilcoxon signed-rank test was used to compare glycan traits, and significant changes are indicated with bold text (significance level α < 0.0012 after Bonferroni correction for 42 tests per time point). The direction of each change is marked with an arrow (up for an increase, down for a decrease). The formula for trait calculation are given in Supplementary Table S2. Lastly, the values listed for galactosylation and sialylation are per antenna.
