# Supplementary material for: Pregnancy-associated serum N-glycome changes studied by high-throughput MALDI-TOF-MS
Source: Sci Rep. 2016 Apr 14;6:23296. doi: 10.1038/srep23296 (PMC4831011; doi:10.1038/srep23296)
Supplement: Supplementary Information [file srep23296-s1.pdf]

Supplementary Information for "Pregnancy-associated serum *N*-glycome changes studied by high-throughput MALDI-TOF-MS"

Bas C. Jansen[1], Albert Bondt[1,2,3], Karli R. Reiding[1], Emanuela Lonardi[1], Coen J. de Jong[1], David Falck[1], Guinevere S.M. Kammeijer[1], Radboud J.E.M. Dolhain[2], Yoann Rombouts[1,3,4], Manfred Wuhrer[1]

[1]Center for Proteomics and Metabolomics, Leiden University Medical Center, 2300 RC Leiden, The Netherlands
[2]Department of Rheumatology, Erasmus University Medical Center, 3000 CA Rotterdam, The Netherlands
[3]Department of Rheumatology, Leiden University Medical Center, 2300 RC Leiden, The Netherlands
[4]Univ. Lille, CNRS, UMR 8576, UGSF, Unité de Glycobiologie Structurale et Fonctionnelle, F 59 000 Lille, France

Corresponding author: Manfred Wuhrer, m.wuhrer@lumc.nl, Tel. +31-71-5268744

Table of Contents

Supplementary Figure S1: Average TSNG spectrum of the 3rd trimester S-2

Supplementary Figure S2: Analyte QC values S-4

Supplementary Figure S3: Changes in glycoforms during and after pregnancy S-5

Supplementary Figure S4: Relative abundance and standard deviation of technical replicates S-6

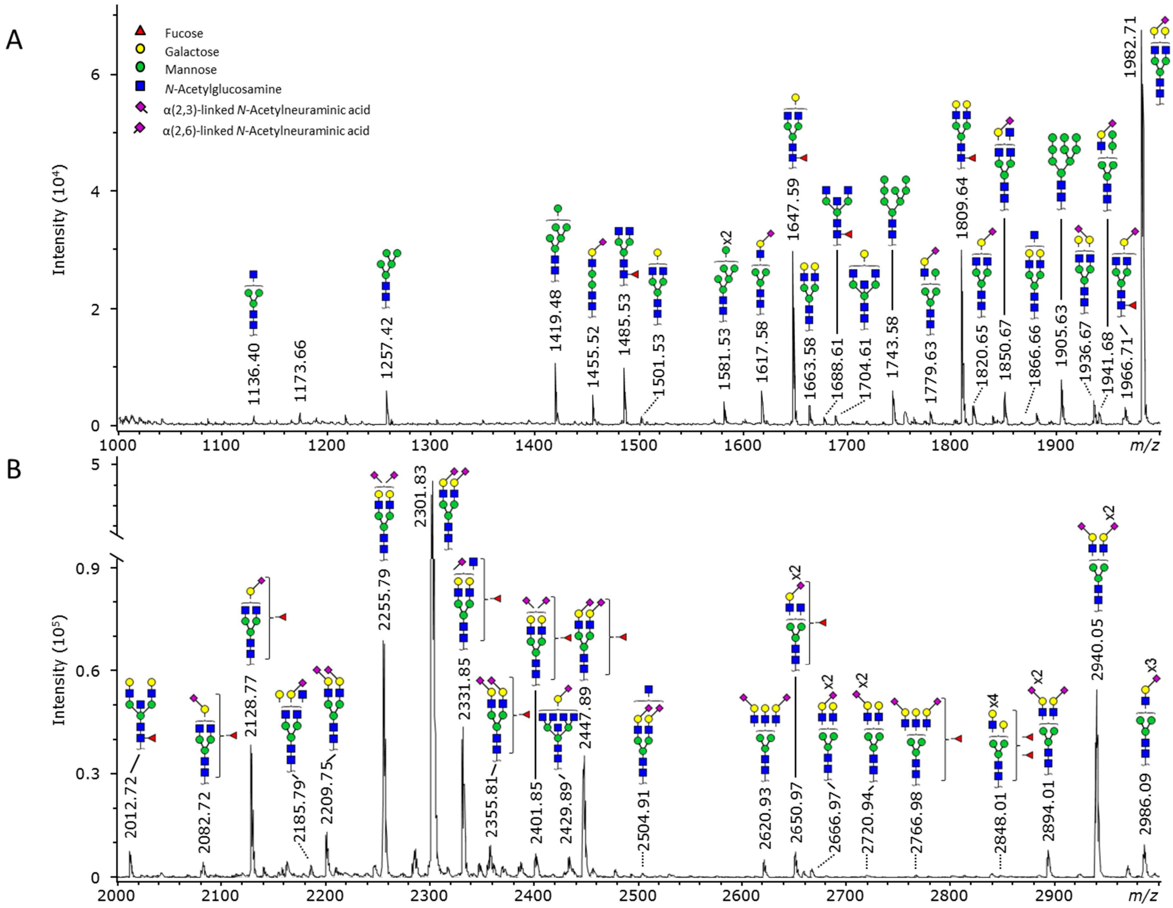
A
Fucose
Galactose
Mannose
N-Acetylglucosamine
α(2,3)-linked N-Acetylneuraminic acid
α(2,6)-linked N-Acetylneuraminic acid
Intensity (10⁴)
1136.40
1173.66
1257.42
1419.48
1455.52
1485.53
1501.53
1581.53
1617.58
1647.59
1663.58
1688.61
1704.61
1743.58
1779.63
1809.64
1820.65
1850.67
1866.66
1905.63
1936.67
1941.68
1966.71
1982.71
m/z
B
Intensity (10⁵)
2012.72
2082.72
2128.77
2185.79
2209.75
2255.79
2301.83
2331.85
2355.81
2401.85
2429.89
2447.89
2504.91
2620.93
2650.97
2666.97
2720.94
2766.98
2848.01
2894.01
2940.05
2986.09
m/z

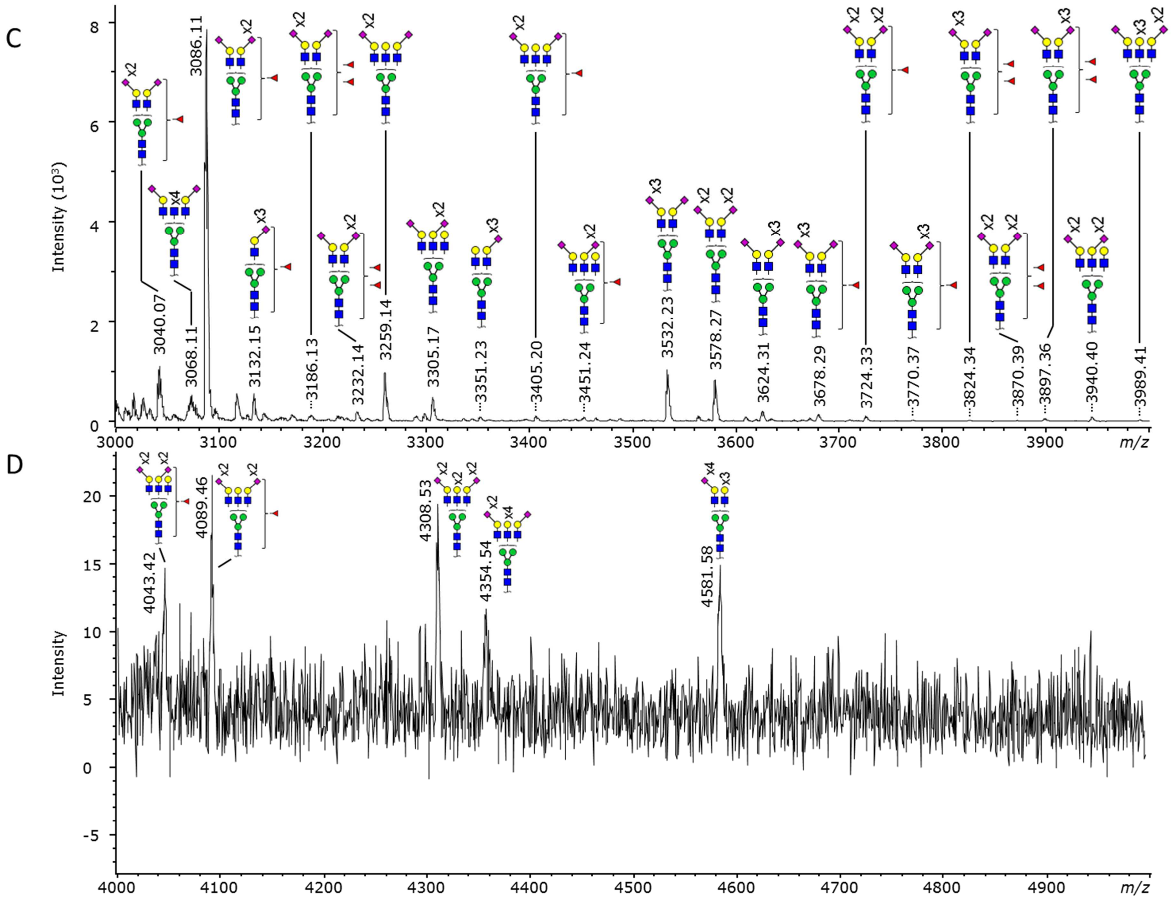

Supplementary Figure S1. Average TSNG spectrum of the 3rd trimester. MALDI-TOF-MS spectrum *of* ethyl esterified released glycans from TSNG, measured in RP mode. (**A**) Spectrum from *m/z* 1,000.00 to 2,000.00. (**B**) Spectrum from *m/z* 2,000.00 to 3,000.00. (**C**) Spectrum from *m/z* 3,000.00 to 4,000.00. (**D**) Spectrum from *m/z* 4,000.00 to 5,000.00. The displayed spectrum is annotated with all observed glycan structures. For high-mannose and hybrid structures, the proposed structures are based on the synthesis pathway.[2] Glycans that are mainly contributed by immunoglobulin G (e.g. H3N4F1, H4N4F1 and H5N4F1) have been well characterised.[3] The localisation of galactose to the specific antennae cannot be elucidated by MALDI-MS. Glycans that contain an *N*-acetylhexosamine additional to the core or LacNAc units (e.g. H5N5F1S1 and H5N5S1) can either be bisected or contain a truncated antenna. Two of the major plasma glycoproteins, IgA and IgM, are known to contain diantennary bisected glycans.[4,5] Antenna fucosylation has been observed on triantennary glycans, for example in α1-acid glycoprotein.[6] However, there are other highly abundant glycoproteins with fucosylated trianntenary structures for which the fucose linkage is unknown. Lastly, sialic acid linkages in the spectrum are based on a derivatisation technique that creates a unique mass for α2,3-linked and α2,6-linked sialic acids.[7] A full list of all compositions that were extracted from this sample can be found in **Supplementary Table S1**.

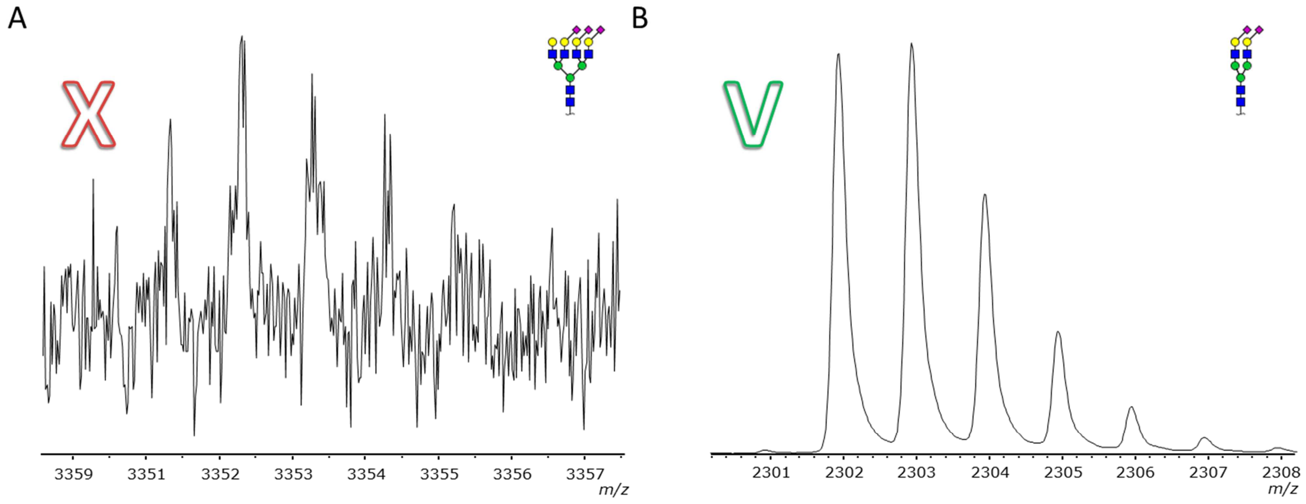

Supplementary Figure S2. Analyte QC values. (**A**) Example of a MALDI-TOF-MS spectrum region around an analyte that was discarded because the average over all spectra was a QC value of $1.5{\cdot}10^{-4}$ and a S/N value of 5.1. (**B**) Example of a MALDI-TOF-MS spectrum region around an analyte that was retained because the average over all spectra was a QC value $3.8{\cdot}10^{-8}$ of and a S/N value of 3440.6.

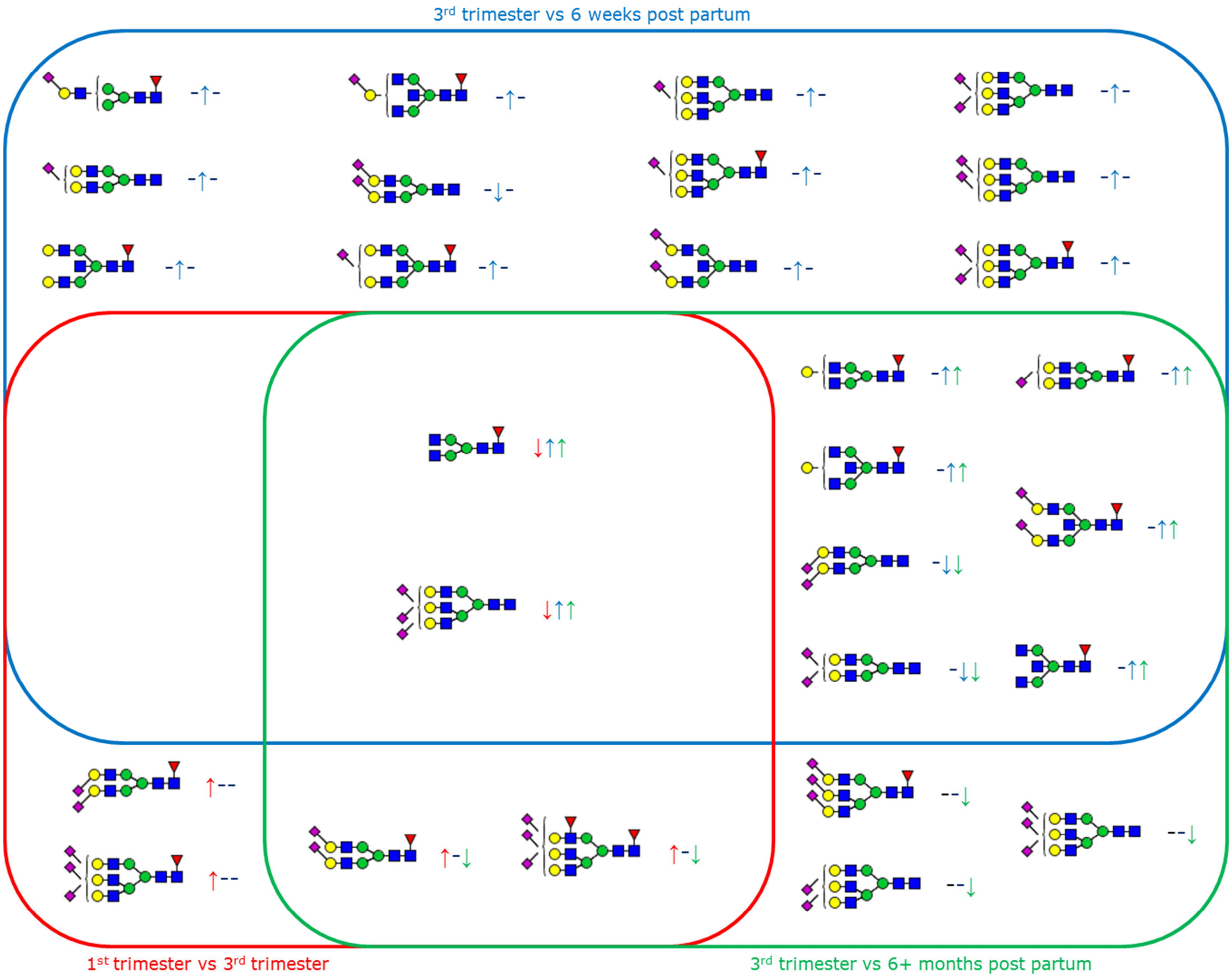

Supplementary Figure S3. Changes in glycoforms during and after pregnancy. Glycoforms that significantly change throughout pregnancy are shown in a Venn diagram. Traits are clustered based on their significance in the performed statistical tests, for pregnancy (1st trimester versus 3rd trimester), short term recovery (3rd trimester versus 6 weeks postpartum) and the long term recovery (3rd trimester versus more than 6 months postpartum). The direction of the change is marked by arrows, an upwards arrow indicates that a trait is increased while a downwards arrow indicates that a trait is decreased. Fucosylation is generally depicted as core fucosylation, although for fucosylated triantennary glycans carrying α2,3-linked sialylation the fucose is most likely on the antenna.[1] Two tables listing all tested glycoforms and a summary of the above results are included in the **Supplementary Table S5** and **S6**.

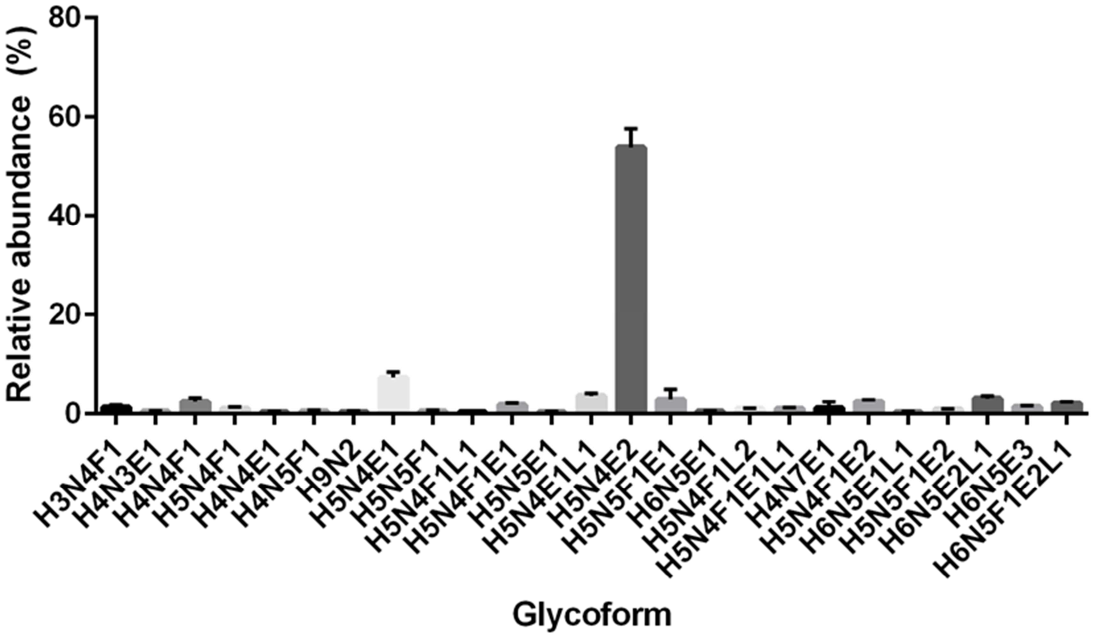

Supplementary Figure S4. Relative abundance and standard deviation of technical replicates. The relative abundance and standard deviation of the 25 most abundant glycoforms that were measured in the 10 technical replicate samples is displayed here. The graph illustrates that there is a low amount of technical variation observed in the technical replicates, which is even further reduced by using the derived traits as a means to examine the biological effects. For instance, the derived trait 'galactosylation of all glycans' shows an standard deviation of only 0.8%.

References

1. Clerc, F.; Reiding, K. R.; Jansen, B. C.; Bondt, A.; Kammeijer, G. S. M. Human plasma protein N-glycosylation. *Glycoconjugate J.* **2015,**
2. Kornfeld, R.; Kornfeld, S. Assembly of asparagine-linked oligosaccharides. *Annu Rev Biochem* **1985,** 54, 631-664.
3. Fujii, S.; Nishiura, T.; Nishikawa, A.; Miura, R.; Taniguchi, N. Structural heterogeneity of sugar chains in immunoglobulin G. Conformation of immunoglobulin G molecule and substrate specificities of glycosyltransferases. *J Biol Chem* **1990,** 265, 6009-6018.
4. Arnold, J. N.; Wormald, M. R.; Suter, D. M.; Radcliffe, C. M.; Harvey, D. J.; Dwek, R. A.; Rudd, P. M.; Sim, R. B. Human serum IgM glycosylation: identification of glycoforms that can bind to mannan-binding lectin. *J. Biol. Chem.* **2005,** 280, 29080-29087.
5. Mattu, T. S.; Pleass, R. J.; Willis, A. C.; Kilian, M.; Wormald, M. R.; Lellouch, A. C.; Rudd, P. M.; Woof, J. M.; Dwek, R. A. The Glycosylation and Structure of Human Serum IgA1, Fab, and Fc Regions and the Role of N-Glycosylation on Fcα Receptor Interactions. *J. Biol. Chem.* **1998,** 273, 2260-2272.
6. Dage, J. L.; Ackermann, B. L.; Halsall, H. B. Site localization of sialyl Lewisx antigen on α1-acid glycoprotein by high performance liquid chromatography-electrospray mass spectrometry. *Glycobiology* **1998,** 8, 755-760.
7. Reiding, K. R.; Blank, D.; Kuijper, D. M.; Deelder, A. M.; Wuhrer, M. High-throughput profiling of protein N-glycosylation by MALDI-TOF-MS employing linkage-specific sialic acid esterification. *Anal. Chem.* **2014,** 86, 5784-5793.
